# Fundamentals of HDX-MS

**DOI:** 10.1042/EBC20220111

**Published:** 2023-03-29

**Authors:** Vanesa Vinciauskaite, Glenn R. Masson

**Affiliations:** Division of Cellular and Systems Medicine, School of Medicine, University of Dundee, Dundee, DD1 9SY, United Kingdom

**Keywords:** HDX-MS, LC-MS, molecular interactions

## Abstract

Hydrogen deuterium exchange mass spectrometry (HDX-MS) is becoming part of the standard repertoire of techniques used by molecular biologists to investigate protein structure and dynamics. This is partly due to the increased use of automation in all stages of the technique and its versatility of application—many proteins that present challenges with techniques such as X-ray crystallography and cryoelectron microscopy are amenable to investigation with HDX-MS. The present review is aimed at scientists who are curious about the technique, and how it may aid their research. It describes the fundamental basis of solvent exchange, the basics of a standard HDX-MS experiment, as well as highlighting emerging novel experimental advances, which point to where the field is heading.

## Introduction

The increasing use of hydrogen deuterium exchange mass spectrometry (HDX-MS) can be attributed to the broad range of proteins, which are suited to the technique and its relatively low technical and theoretical barriers. One key aspect of the renaissance of HDX-MS is its ability to provide structural information on many challenging targets for drug discovery that represent a step-change in ambition from the well-ordered easily crystallizable small proteins that readily provided insight into structural–activity relationships between proteins and ligands. Targeting very large proteins, which may be embedded in membranes, contain post-translational modifications (PTMs) or regions of intrinsic disorder, and the arrival of biopharmaceuticals, accelerated the technological progress in both mass spectrometry instrumentation, and software to provide a much more accessible process of sample preparation, data acquisition, and data analysis [1–13]. Ten to 15 years ago, HDX-MS experiments were conducted by specialists with homemade fluidic systems and software developed in-house. Now, most HDX-MS experiments are automated to some degree using commercially available systems.

A major attraction of HDX-MS is that in addition to providing structural data, HDX-MS can be used to investigate protein dynamics by monitoring solvent exchange over time. The data obtained from HDX-MS are highly complementary to orthogonal structural, biophysical, and biochemical techniques, as well as molecular dynamic simulations [14–21]. The present review provides the reader an introduction to HDX-MS and how it can be used to unveil both protein structure and dynamics. Topics ranging from the principle of solvent exchange, detection, and data acquisition, as well as experimental designs, data analysis, and the future of the technique are discussed.

## Fundamentals of solvent exchange

**Figure 1:**
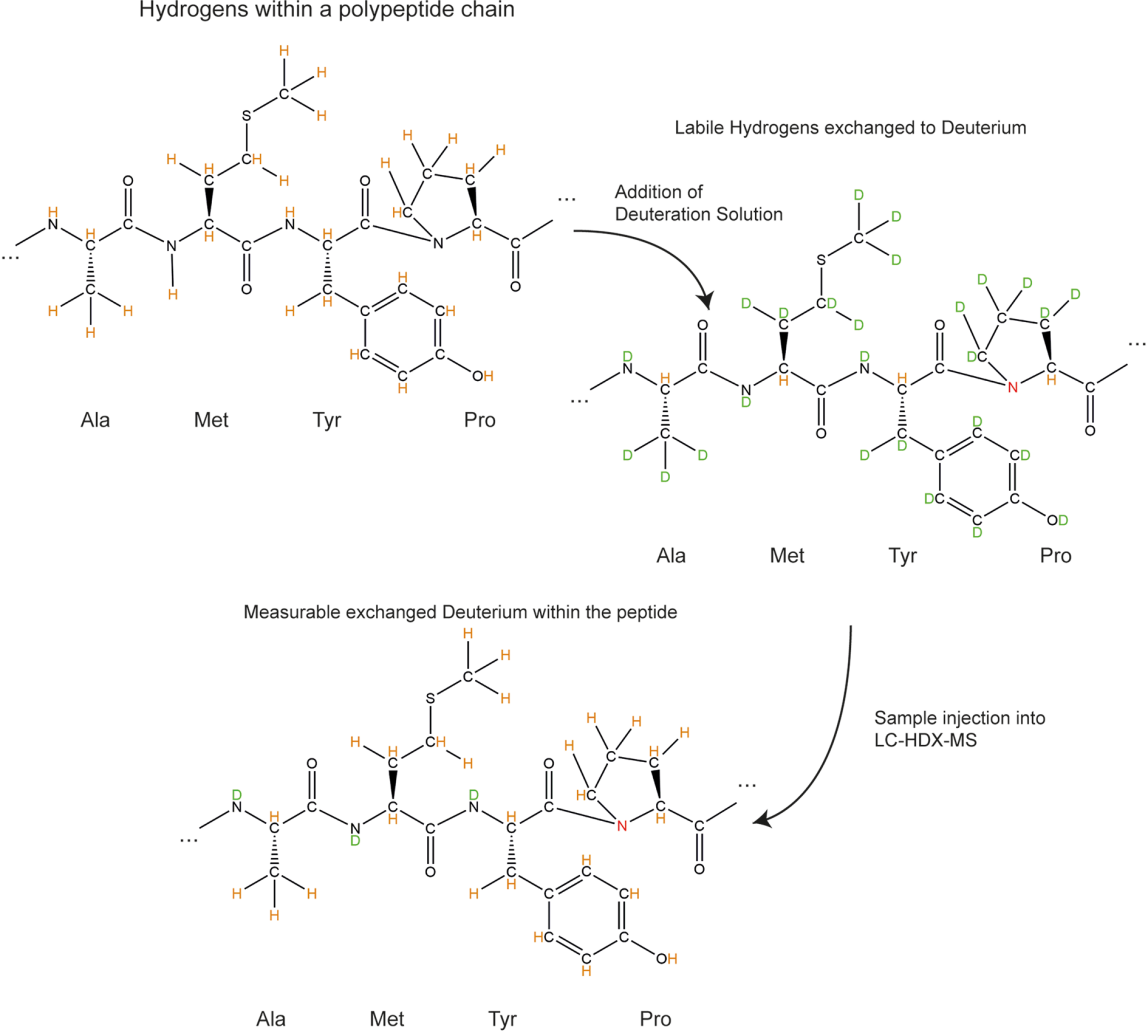
A polypeptide chain composed of alanine, methionine, tyrosine, and proline Upon the addition of a deuterium solution, the backbone amide hydrogens as well as many functional group hydrogens are exchanged to deuterium (only α-hydrogens are not exchanged in a typical protein HDX experiment). The exact rate of side chain exchange is variable, and some may not exchange in the time course of the experiment. However, due to back exchange, upon sample injection into the liquid chromatography (LC) system, functional group deuterium exchanges back to hydrogen, and only the backbone amide deuterium remains intact. As shown in the figure, exchange in proline is unviable for measurements due to the backbone amide lacking a hydrogen.

At its core, HDX-MS measures *the rate of solvent exchange*—the phenomenon whereby atoms comprising the solvent are swapped for atoms of the protein (and *vice versa*). HDX-MS tracks solvent exchange by exposing the hydrogens within a protein molecule to a solvent containing a heavier hydrogen isotope—deuterium. The mass of a deuterium isotope is approximately 2.0141 Da, which is approximately double the mass of hydrogen (∼1.0080 Da), meaning that a solvent exchange event results in a small increase in the mass of a molecule. A 1 Da increase in mass is miniscule when compared with the mass of a protein; however, detecting and locating this increase in mass is eminently achievable with modern mass spectrometry equipment. By exposing proteins to a deuterated solvent for a defined period, quenching the solvent exchange reaction, and then measuring the increase in mass with a mass spectrometer, it is possible to determine solvent uptake progress over time. While solvent exchange is possible on all the labile hydrogens of a protein (i.e., any of the -NH, -OH, or -SH groups), only the peptide backbone amide hydrogens of the protein are tracked in a typical HDX-MS experiment (see Figure 1). There are several benefits to this—peptide backbone hydrogens are uniformly distributed throughout the protein (save for proline-rich areas) and form secondary structure elements through hydrogen bonding.

The rate of solvent exchange is influenced by numerous factors—the temperature and pH of the solvent, the chemistry of the amino acid, solvent accessibility, and the hydrogen-bonding network [22]. Each amino acid has an associated intrinsic exchange rate, called the chemical rate constant (*k*_ch_)—the rate of exchange that a given amino acid would have in the absence of secondary or tertiary protein structure—which is influenced by neighboring amino acids, temperature, pH, pressure, and ionic strength [23,24]. As solvent exchange progresses through base catalysis (through a hydroxide ion (OH^−^)) or acid catalysis (through a hydronium ion (H_3_O^+^)), the chemical rate constant for any given backbone amide hydrogen is given as:

$$k_{\mathrm{ch}}=k_{\mathrm{int},\mathrm{acid}}[H_{3}O^{+}]+k_{\mathrm{int},\mathrm{base}}[{\mathrm{OH}}^{-}]+k_{\mathrm{int},\mathrm{water}}[H_{2}O]$$

For a solvent exchange reaction to occur, a hydrogen bond must form between the atom in question and the solvent when this is possible the peptide is deemed to be in an ‘open’ state [25,26]. Proteins are not static molecules; they are constantly in motion and there will be local transitions from an ‘open’ state to a ‘closed’ state. The description of exchange kinetics can be expanded with the rate of ‘opening’ as *k*_op_, and the reverse, “closing,’ as *k*_cl_:

$$\text{N}-{\text{H}}_{\mathrm{closed}}\overset{k_{\mathrm{op}}}{\underset{k_{\mathrm{cl}}}{⇄}}\text{N}-{\text{H}}_{\mathrm{open}}\overset{k_{\mathrm{ch}}}{\to }\text{N}-{\text{D}}_{\mathrm{open}}\overset{k_{\mathrm{cl}}}{\underset{k_{\mathrm{op}}}{⇄}}\text{N}-{\text{DH}}_{\mathrm{closed}}$$

Additionally, although exchange is reversable in the open state, in the experimental design, it is assumed that [deuterium] >> [hydrogen], forcing the exchange step to be unidirectional. From this, the rate of exchange, *k*_ex_, is described as:

$$k_{\mathrm{ex}}=\frac{\left(k_{\mathrm{op}}k_{\mathrm{ch}}\right)}{\left(k_{\mathrm{cl}}+k_{\mathrm{ch}}\right)}$$

Finally, two kinetic regimes dominate protein dynamics, EX1 and EX2. First, EX1—where *k_ch_ >> k*_cl_, and thus *k*_ex_ ≈ *k*_op_, meaning that each slow ‘opening’ event has a complete exchange reaction. This creates a labeled population of the protein during each exposure event, and when using mass spectrometry that can be easily visualized as a bimodal distribution of the isotopic spectrum. In an EX2 dynamic regime, where *k*_ch_* << k*_cl_, each opening event provides an ‘opportunity’ for exchange to occur. Most structured regions of a protein will be in a ‘closed’ state with inaccessible hydrogens with EX2 dynamics. Deuteration of unfolded, flexible, and dynamic regions switch between open and closed state fast and exchange occurs on the millisecond timescale. More structured regions may require anything between minutes to hours and days to undergo such transitions. Under native conditions, it is believed that EX2 kinetics are predominant, but large domain reorganizations may result in EX1 kinetics, and ‘mixed’ EX1/EX2 regimes are observed [27,28].

## Measurement of HD exchange

Due to the sensitivity of exchange rates to perturbations in temperature and solvent conditions, it is crucial for multiple observations to be taken. Therefore, HDX experiments should be repeated using both biological and technical replicates. Additionally, due to the kinetic nature of uptake mechanics, multiple timepoints should be conducted to ensure a complete sampling of the peptide backbone. For a full guide on the minimum standards of HDX-MS experiments, including data collection and reporting, see [29]. To initiate the HDX reaction, the protein of interest is rapidly diluted with a labeling solution that contains a large excess of D_2_O to ensure that the exchange reaction is unidirectional. The exchange reaction can be slowed many orders of magnitude by the addition of an acidic (pH 2–3) quench solution [30]. The sample can be directly injected to a LC system, which is connected to a mass spectrometer or frozen in liquid nitrogen and stored at –70 to 80°C [31]. Additionally, nondeuterated samples are acquired to establish a mass for each peptide (as no exchange reaction takes place in nondeuterated samples, time point experiments for the nondeuterated samples are not required).

## HDX-MS experiment design

Depending on the research question, there are multiple ways to conduct an HDX-MS experiment. The three main methods are bottom-up, top-down, and middle-down HDX-MS experiments. This review aims to briefly describe these methods—reading the recommended literature for each method is highly advised for a more in-depth understanding of the techniques.

**Figure 2:**
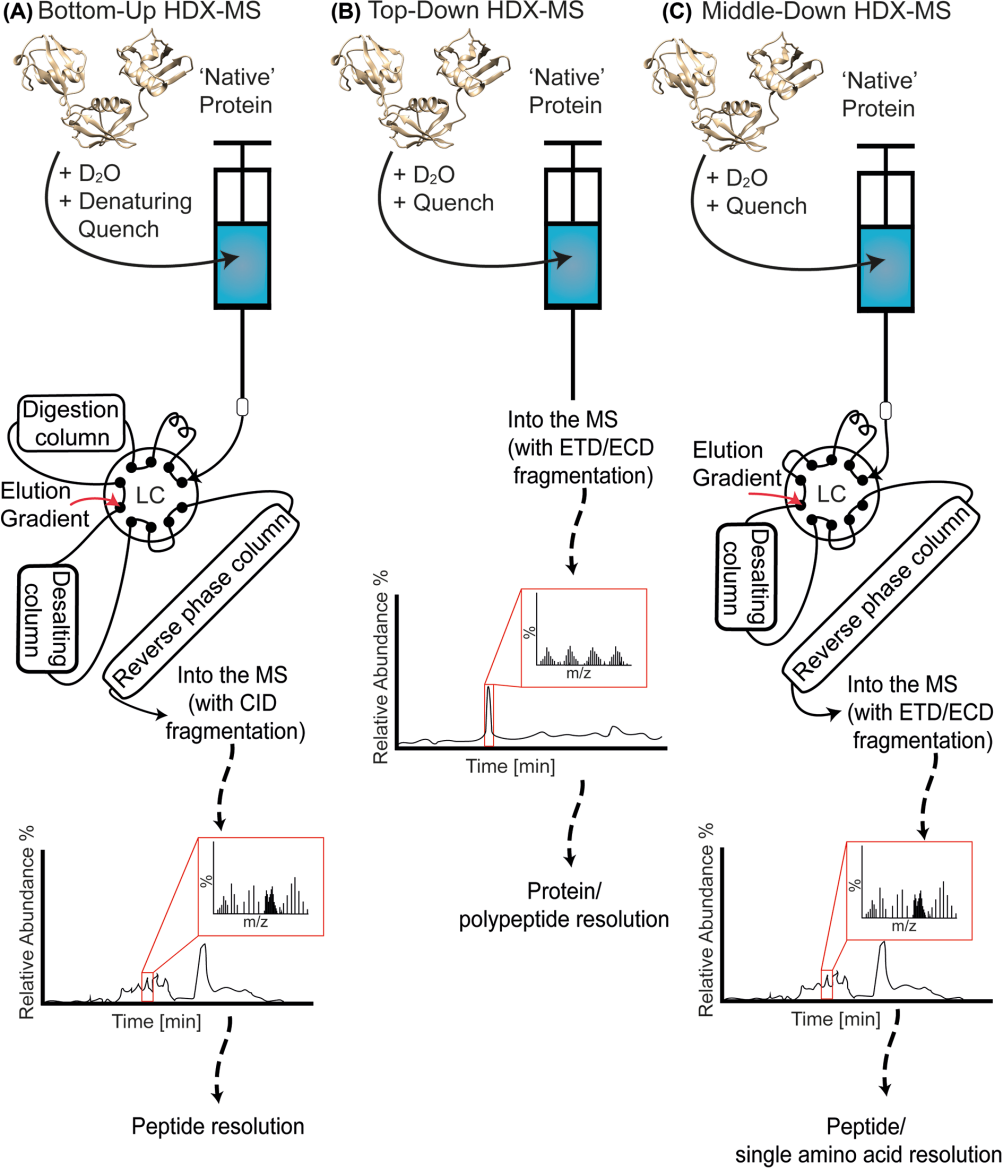
Representation of common HDX-MS workflows Schematic overview of (**A**) bottom-up-, (**B**) top-down-, and (**C**) middle-down HDX-MS experiment. Note that only in bottom-up and middle-down HDX-MS experiments, local resolution may be achieved. The polyubiquitin-B (P0CG47) model was obtained from Alphafold Protein Structure Database [89].

The most frequently used experimental design is bottom-up HDX-MS, which provides a peptide level resolution of exchange kinetics (Figure 2A). Bottom-up HDX identifies ordered and disordered regions of a protein, interaction interfaces, and structural rearrangements accompanying or protein–ligand interactions [32–35]. Frequently, bottom-up HDX-MS is used comparatively to determine the structural differences between a protein in two states, such as wild-type and mutant, unmodified and post-translationally modified, and with and without binding partner—it is also widely used to map antibody and nanobody epitopes. When using HDX-MS to investigate binding partners, such as antibodies or compounds, it is crucial to design the experiment to ensure occupancy of the protein—especially for weakly binding complexes (see Text Box 1).

Text Box 1 **Ligand occupancy**

As with any bulk measurement, it is necessary to ensure homogeneity of the sample being measured. HDX-MS can measure weak interactions (with low millimolar *K*_d_), but necessary steps are required to ensure the protein-binding site remains occupied. If this is not considered, only a population of the protein will be bound to the ligand, causing heterogeneity, with the possibility of a pseudo-EX1-like profile to emerge at the binding site. For an in-depth discussion of the necessary considerations required when designing an HDX-MS experiment with weak binders, see [90].

First, the sample is labeled with a D_2_O solution for several predetermined time points and then a quench solution is added to the reaction mix and injected onto a liquid-chromatography mass spectrometry (LC-MS) system (or alternatively rapidly frozen in liquid nitrogen and stored at –80°C). It is important to note that the quench solution not only slows the exchange reaction through dropping the pH, it also typically contains a chaotropic agent (such as urea or guanidinium hydrochloride) to denature the protein and enable rapid proteolysis downstream (Text Box 2). The sample subsequently undergoes enzymatic digestion using either an online immobilized protease column in the LC-MS, or separately using proteases on beads that are subsequently removed prior to analysis [36]. As previously described, LC steps are typically conducted rapidly at low temperature and low pH [37–39]. Ideally, the enzymatic digestion of the sample generates numerous overlapping peptides, ranging from 5 to 30 amino acids in length, allowing analysis at peptide resolution. Several different proteases are available, such as (but not limited to) nepenthesin, fungal protease type XIII, and the most frequently used—pepsin [40–42]. A key feature of these proteases is their nonspecific cleavage producing many overlapping peptides, and their functionality at the low pH, low temperature, and under high pressure conditions required for an HDX-MS experiment. The digested peptides pass onto a desalting column before being loaded onto a reverse-phase column. Peptides are then eluted through a gradient of increasing acetonitrile, typically lasting less than 15 min. The low temperatures and rapid gradients required for HDX-MS peptide chromatography typically result in poor separation of peptides as they are eluted from the reverse-phase column. This can be overcome through optimization of the elution chromatography, and using ion mobility spectrometry (IMS) [43]. Eluted peptides are then subjected to ionization by electrospray ionization (ESI) [44].

Text Box 2 **Optimization of an HDX-MS experiment**

Prior to conducting an HDX-MS experiment, different nondeuterated conditions are tested to obtain an optimal condition, which provides good resolution and the highest sequence coverage (for middle-down and bottom-up HDX-MS).

To optimize the signal-to-noise ratio as well as to obtain from the MS analysis, various concentrations of the protein are injected into the HDX-MS system to optimize the signal. The required amount may depend on the sensitivity of the mass spectrometer, nonetheless 5 pmol of protein is recommended as the starting point.

During the optimization of the protein, different chaotropic agents in the quench solution may be screened, of which guanidinium hydrochloride and urea are the most common. High concentrations of either denaturant will unfold α-helices and β-sheets, which will provide more access to the cleavage sites for the protease. As such different concentrations of each denaturant may be screened, heavily depending on the topology of the protein. Concentrations between 1.6 M and 6 M guanidinium chloride as well as 2 M to 8 M urea may be screened to obtain the most varied number of peptides.

Furthermore, it is important to be aware of ‘Carry-Over’—the term given to the peptides retained on the fluidics system from the sample immediately preceding the current sample. These peptides may impact subsequent analysis as they typically present as less deuterated (due to the increased exposure to solvent and thus increased back exchange). Running a buffer blank through the fluidics system after the sample and observing if peptides are still in high abundance (>10% of the previous run) is recommended, as such optimizing washes of the proteolytic column and quench are required to combat this.

HDX-MS presents unique challenges for MS/MS studies [45]. To determine peptide sequences, the nondeuterated samples’ peptides are typically fragmented using collision-induced dissociation (CID) to produce *b* and *y* ions. Conducting MS/MS studies on deuterated peptides would be highly advantageous as it would enable an amino acid resolution localization of deuterium incorporation, enabling a better insight into solvent exchange reactions. However, it is not possible to accurately fragment peptides by CID due to the phenomenon of ‘scrambling’—the randomization of the deuterium signal on peptides during high-energy fragmentation methods [43]. ‘Soft’ fragmentation methods such as electron transfer dissociation (ETD) and electron capture dissociation (ECD) are compatible with HDX-MS and can bring the resolution from peptide level to single residue—but may require several controls to ensure scrambling is not occurring [43,46–50]. The use of ETD/ECD is not yet common, primarily due to the increased complexity of experimentation and data analysis.

Additionally, a maximally deuterated (D_max_) sample may be acquired as well to correct deuteration levels for back exchange (Text Box 3) [23,51].

Text Box 3 **Back exchange and D_max_**

One key consideration for HDX-MS experiments is the process of back-exchange. This occurs when the deuterium-labeled protein is exposed to a protiated solvent and exchange occurs, resulting in deuterium on the protein being swapped back for a hydrogen. This process is undesirable as the label is essentially lost, producing an underestimation of exchange rate, and this may lead to misinterpretation of results. To prevent back-exchange from occurring, HDX-MS experiments are conducted rapidly (chromatography is conducted in minutes rather than days or hours), at low (∼0°C) temperature and in acidic (∼pH 2) environments. This places unique constraints on the experimental design.

Back-exchange and can be corrected for when a D_max_ control is used. A D_max_ control is a sample where every single backbone amide is deuterated. To obtain a D_max_ value, the protein of interest is incubated with a chaotropic agent and is digested with a protease. Proteolytic peptides are then separated from the protease and incubated with D_2_O to complete deuteration—exchange will be far more rapid with the unstructured peptides when compared with the folded protein. Correcting for back-exchange is good practice, as it ensures that there is not excessive back-exchange with certain peptides, which may lead to an incorrect interpretation of results—however, it is not critical for all experiments.

Intact or top-down HDX-MS experiments analyze the global changes of a protein (Figure 2B). The sample is labeled with deuterium for predetermined time points, the reaction is quenched by a quench solution (with no chaotropic agents) and the sample is injected directly onto the ESI source. Alternatively, microfluidic, or capillary chambers may be employed to label the protein over time, resulting in a continuous sampling of timepoints as the protein is injected into the source [52,53]. The sample may then undergo mild fragmentation by either an ECD or ETD to cleave labile interdomain bonds, providing additional ‘domain’ level resolution [54–56]. While top-down experiments will not provide local resolution of mass shifts, the technique is utilized to compare global deuterium incorporation rates and observe interspecies differences that may occur, such as the influence of post-translational modifications, as well as it will aid in characterizing conformationally heterogenous samples [57–59].

Middle-down experiments are less common and are a hybrid of the forementioned methods (Figure 2C). The sample preparation is the same as for bottom-up-HDX, with the exception that the enzymatic digestion is done offline by a specific enzyme that produces long peptides. The peptides are then subjected to ESI and ETD/ECD. The middle-down approach leads to longer peptides obtained, yet the sequence resolution is expanded due to the fragmentation method. The method may be of interest to study short-sequenced proteins, where high data resolution may be obtained from a few peptides [60,61]. As specific enzymes must be used for this technique, it is advised to check enzymatic digestion patterns with available bioinformatic tools to observe the compatibility between the sample and digestion [62].

## HDX-MS data analysis and interpretation

The first step of data interpretation is to identify the sequences and PTMs on the peptides obtained from nondeuterated spectral data by a database search engine, either via commercial software provided by the manufacturer of the mass spectrometer, such as ProteinLynx Global Server (PGLS) by Waters, PaSER (Bruker), Proteome Discoverer (Thermo Fisher Scientific); or by license-free software as MASCOT or Comet [63,64]. The search engines utilize distinct packages to identify peptides that are well summarized elsewhere [65]. It is important to ensure that the sequence of the protease used in the experiment is included within the search database, and additionally, if the protein has been produced recombinantly, common contaminants from the expression system are also included. It is recommended to check digestion profiles of proteases, especially the nonspecific pepsin, to mitigate the number of falsely identified peptides [66].

Both commercial and noncommercial HDX-MS downstream data analysis software exist to measure the mass shift of the isotopic envelope in a charge state (summarized by [67]). Before individual peptides are chosen for data analysis, certain criteria should be applied. Peptides obtained from bottom-up and middle-down pathways may be rejected from further analysis if there are large deviations between repeats of retention time, intensity and/or mobility (if applicable) criteria between repeats [68]. Generally, the obtained sequence coverage should reach over 90% for sufficient identification of protein topology. Briefly, programs aim to match the peptide ion envelope/cluster in a specific mass, retention time, (if obtained) mobility drift range of a deuterated sample to the preprocessed nondeuterated sample. The ion envelope peak centroids or the deconvolution of the ion cluster are used to interpolate the level of deuteration, as the shift of the cluster to a higher mass in comparison to the nondeuterated sample indicates the incorporation of deuterium. The mean mass shift (of all replicates) at a given time point can be indicated as a percentage of deuterium incorporation (where the measured D_max_ = 100%), the mass shift is observed in Daltons, or the number of deuterons incorporated into the peptide.

## Data interpretation

Spectral changes observed during HDX data analysis are driven by the incorporation of deuterium, shifting the spectral isotopic envelope to higher values for any given charge state. One unique property of HDX-MS is that the shape of the spectra can provide useful information on protein dynamics.

**Figure 3:**
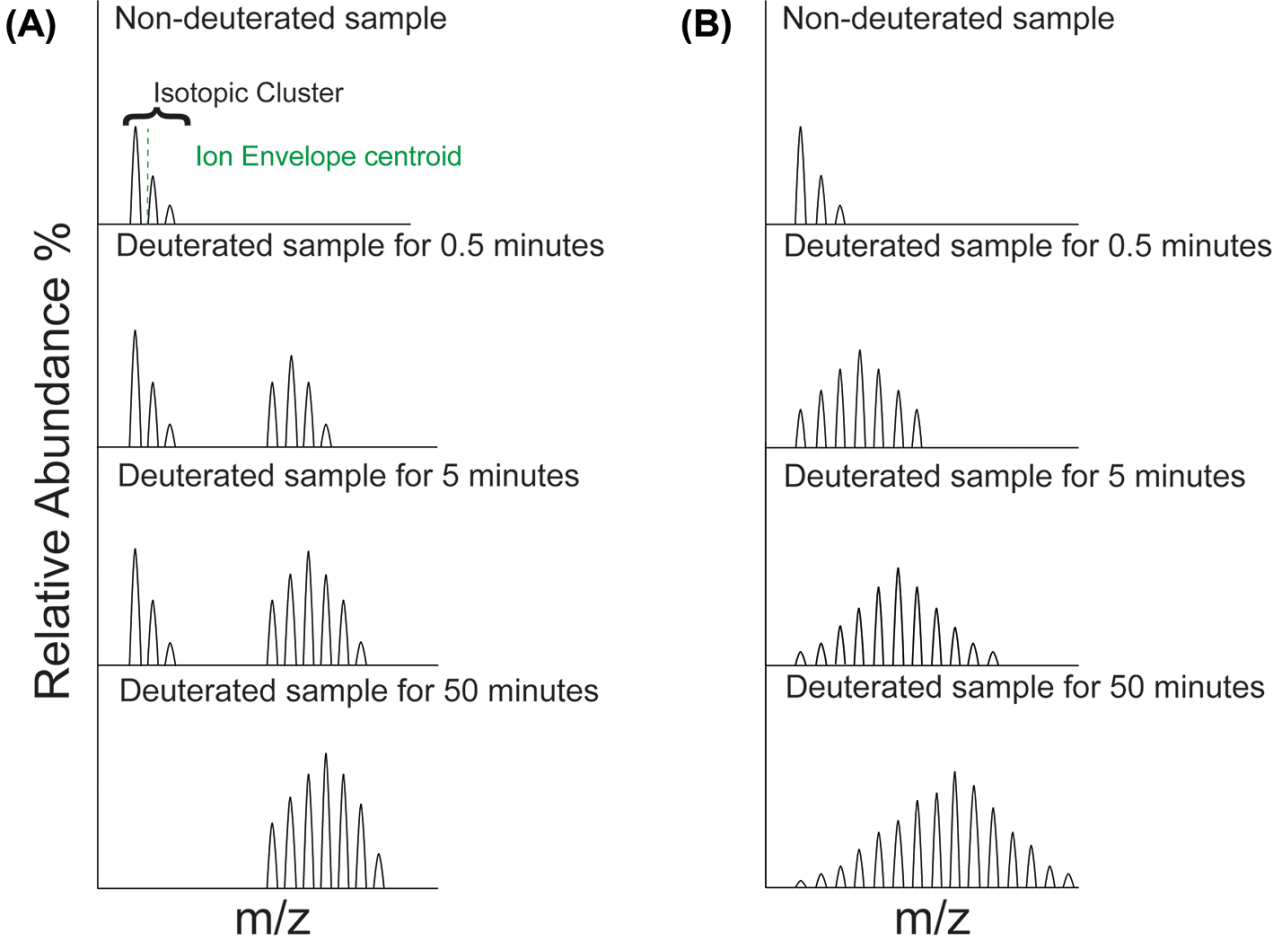
Overview of isotopic clusters observed during a continuous HDX-MS experiment, at different time points Specifically, (**A**) EX1 or (**B**) EX2 kinetics are shown.

A peptide undergoing EX1 kinetics manifests as multiple mass shifts due to deuteration, where multiple isotopic envelope species are observed (Figure 3A). It is important to note, however, this may also be the result of conformational heterogeneity within the sample (such as the presence of precipitation). In top-down-HDX-MS, these shifts correlate to different protein conformation species, while in bottom-down-HDX-MS, these shifts suggest that there are folding/unfolding events occurring during the labeling reaction, and while these folding events are possible during deuteration, it is important to confirm them. It is crucial to first observe if such spectra are the result of carry-over from the previous experimental run, which can be eliminated by optimized washing protocols in LC-MS [69].

**Figure 4:**
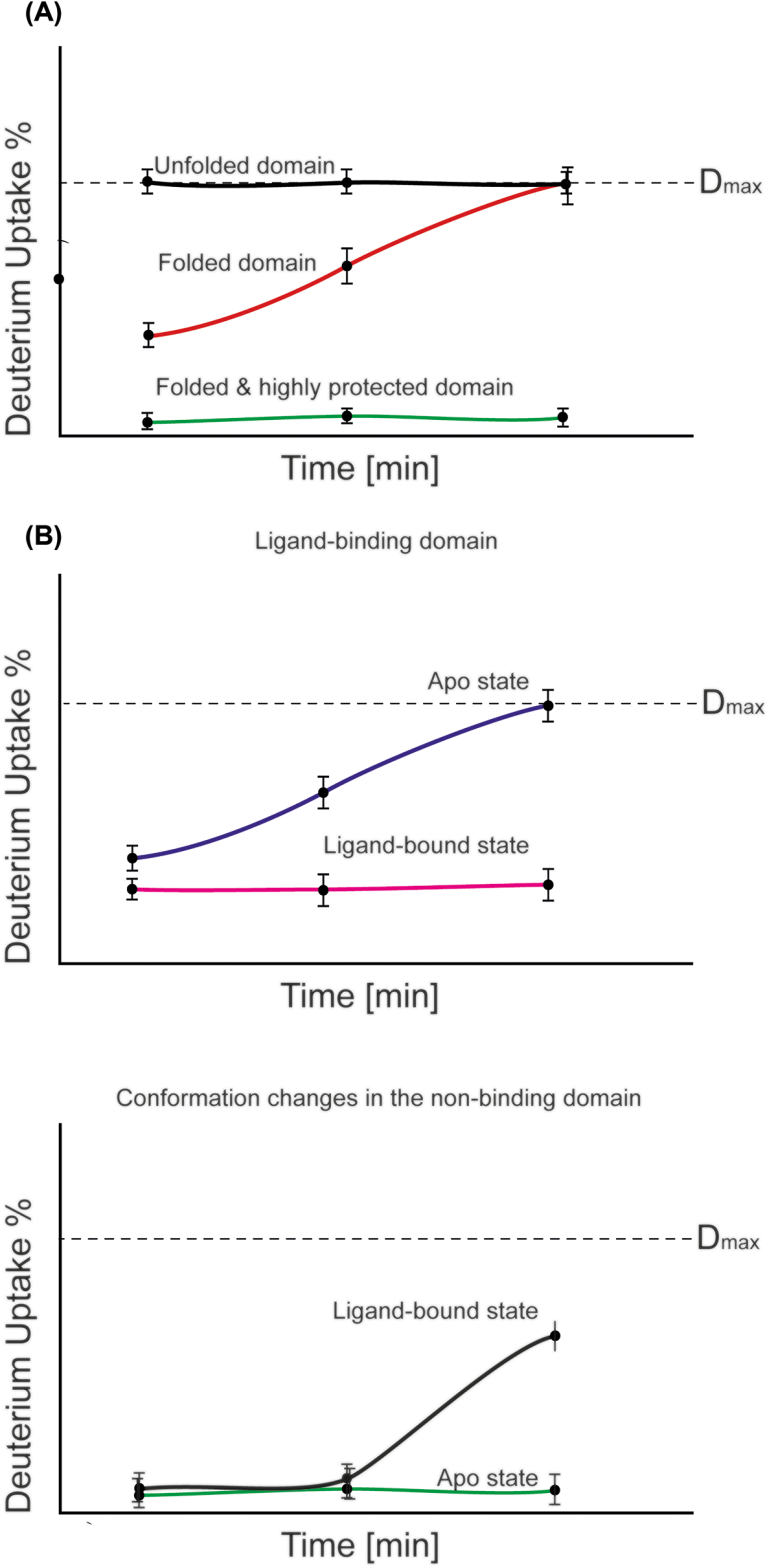
Deuterium uptake plots over time (**A**) Commonly observed deuteration patterns of unfolded, folded as well as protected domains. Upon interaction studies (**B**), the protection against deuteration is observed in the ligand-binding domain and additional domains may undergo increased or decreased (as shown) protection.

Pulse-labeling experiments should be conducted to confirm that the refolding events do occur (Text Box 4). With this experiment, the stability of a protein is observed over time, and this may necessitate further time point experiments. Additionally, exchange in a folded region will take much slower and the observed deuteration may not reach the D_max_ value in the time scale, thus the timescale may be extended with additional time points until the D_max_ is reached (Figure 4A). It is important to double check with pulse-labeling experiments if the observed deuteration patterns are correct. Ligand binding to structured peptides is observed where a binder may incur a change in deuteration for the whole experiment timescale. As well as conformational shifts can be observed.

Text Box 4 **Pulse labeling**

A pulse-labeling experiment is conducted to observe if a protein undergoes structural rearrangements during the labeling experiment, which may impact data obtained from continuous labeling experiments. Gross changes in the protein structure, such as precipitation, may occur due to the instability of the protein within the timescale of the experiment or due to alterations of the environment surrounding the protein, such as changes in pH, temperature, or additional agents in the deuteration solution. Pulse labeling may provide information on the folding and unfolding rate constants that govern the possible intermediate states of the protein as well are crucial to perform when the deuteration labeling is over 100 min if the stability of a protein is unknown.

During a pulse-labeling experiment, the protein is incubated in a deuterium-free solution to mimic a labeling experiment for a period that is equal to a deuteration time point. After a set amount of time, the sample is rapidly labeled with a deuterium solution, followed by a quench solution. It is important to perform the deuteration as fast as possible to observe structural rearrangement transitions from resolved isotope envelopes (if the protein showcases EX1 or mixed EX1/EX2 kinetics). The sample is then compared with the continuously labeled protein, to observe differences between isotopic envelopes, and therefore potential structural differences.

In bottom-up-HDX-MS experiments, EX2 kinetics are commonly observed as a gradual mass shift of deuterium over time as a single isotopic envelope (Figure 3B). As the rate of closing of a protein state is faster than the chemical exchange rate, only a single conformation will be observed. Depending on protein structure in question, the open state be populated in a minute timescale or in case of high protection and slow change to an open state, a bottom-up-HDX-MS experiment may require days of deuteration. If a deuteration reaction occurs longer than an hour, a pulse-label experiment must also be conducted for the aforementioned duration. In unfolded domains, a fast exchange to deuterium may occur, where D_max_ is obtained from a short time point (seconds or less). Proper interpretation of such a region would require conducting the experiment on a millisecond scale (if possible), to observe deuteration patterns, or by manipulating temperature and pH values to slow solvent exchange [70–72].

Typically, a ligand-binding event results in a localized reduction in the deuteration uptake at the interaction site (Figure 4B). The peptide backbone becomes unavailable for solvent exchange as it is already engaged in hydrogen bonding to the ligand. If the ligand induces additional allosteric conformational changes distant from the interaction site, these shifts may also be observed as either increased or decreased deuteration levels over a few (not necessarily all) time points. In this case, additional information is required disentangle the ligand-binding site from allosteric events.

## Data verification

After the initial analysis of HDX-MS data are finished, a robust statistical analysis must be conducted to determine the significance of any changes in peptide deuteration—there are a variety of (non)commercial software tools to facilitate this [73–76]. Typically, the deuteration level of a peptide within a time point is obtained from the shift of centroid mass of the isotopic envelope between the nondeuterated peak centroid and deuterated peak centroid. Deuteration values are then averaged between replicates. Deuteration is viewed as a function of labeling time, where the distribution averages on a given time point are compared with those obtained from other experimental conditions, such as the addition of another protein or ligand. HDX-MS data are commonly presented as individual peptide uptake plots of deuteration over time, where all measured states (unbound and bound with an interacting ligand) are plotted with standard deviations shown. If the structure of a protein is known, structural rearrangements are represented using a three-dimensional model, illustrating increased or decreased amounts of deuteration at a given time point. The HDX-MS community has extended guidelines on how to publish data; and are highly recommended to read through before publishing any HDX-MS results [29].

## Recent advances and future outlooks

Already back in 1980s’, Walter Englander mentioned how important it is to understand ‘*The newly emerging 4^th^ dimension of macromolecular structure, its fluctuations in time’* [26]. While static structures of proteins have been available for decades now, only recent advances in the implementation of dynamics by HDX-MS and other techniques are providing unique information about protein topology and kinetics. In addition to structural and dynamic information, it is observed that HDX-MS can be utilized to observe thermodynamic properties in a protein. Furthermore, with the implementation of automatic sample preparation, HDX-MS has become a high-throughput screening technique. Alongside the resolution revolution within cryoelectron microscopy, the addition of HDX-MS data of the macromolecular assemblies provides unique interaction data that otherwise would be unattainable [77–80]. Currently, advances in HDX-MS focus toward improvements in peptide resolution and data analysis [81–85]. The main drawback of the technique is the intensive manual data analysis, which is the current bottleneck of HDX-MS. Furthermore, many researchers are developing methods to conduct an HDX-MS experiment on the millisecond scale, which would allow for improved characterization of disordered domains. In addition, online double digest columns are being developed—both in-house and, recently, commercially—to improve the number of overlapping peptides and thus, increase the resolution [86]. And lastly, the implementation of ETD/ECD techniques is being more widely used, allowing for single amino acid resolution and true protection factors to be obtained [87,88]. With these advancements, HDX-MS will become a readily available high-throughput technique, which provides high-resolution data of protein ordered and unordered domains.

## Summary

- HDX-MS is used to obtain structural data of proteins and their binding partners to identify the interaction interface and observe the dynamics of the protein over time.
- HDX-MS is highly complementary to other structural techniques, biophysical, and biochemical assays as well as bioinformatic simulation as molecular dynamics.
- With advancing protein digestion techniques, implementation of different fragmentation methods and data analysis software, high-throughput HDX-MS will be capable of routinely obtaining single amino acid resolution data.

## Open Access

Open access for the present article was enabled by the participation of University of Dundee in an all-inclusive *Read & Publish* agreement with Portland Press and the Biochemical Society under a transformative agreement with JISC.

## Abbreviations

CID: collision induced dissociation
Da: Daltons
D_max_: maximum deuteration
ECD: electron collision dissociation
ESI: electron spray ionization
ETD: electron transfer dissociation
EX1: HDX kinetics with an observed bimodel pattern of two differently folded populations
EX2: HDX kinetics with a single uniform population
HDX: hydrogen deuterium exchange
HDX-MS: hydrogen deuterium exchange mass spectrometry
IMS: ion mobility spectrometry
k_ch_: the chemical rate constant
k_cl_: the closing rate constant
k_ex_: the rate of exchange constant
k_op_: the opening rate constant
LC: liquid chromatography
LC-MS: liquid chromatography-mass spectrometry
MS: mass spectrometry
MS/MS: tandem mass spectrometry
PTM: post-translational modification
